# Electrochemical Synthesis of Plasmonic Nanostructures

**DOI:** 10.3390/molecules27082485

**Published:** 2022-04-12

**Authors:** Joshua Piaskowski, Gilles R. Bourret

**Affiliations:** Department of Chemistry and Physics of Materials, University of Salzburg, Jakob Haringer Strasse 2A, A-5020 Salzburg, Austria; joshua.piaskowski@stud.sbg.ac.at

**Keywords:** plasmonics, electrodeposition, templated synthesis, electrochemical synthesis, porous alumina membrane, gold nanorods, on-wire lithography, coaxial lithography

## Abstract

Thanks to their tunable and strong interaction with light, plasmonic nanostructures have been investigated for a wide range of applications. In most cases, controlling the electric field enhancement at the metal surface is crucial. This can be achieved by controlling the metal nanostructure size, shape, and location in three dimensions, which is synthetically challenging. Electrochemical methods can provide a reliable, simple, and cost-effective approach to nanostructure metals with a high degree of geometrical freedom. Herein, we review the use of electrochemistry to synthesize metal nanostructures in the context of plasmonics. Both template-free and templated electrochemical syntheses are presented, along with their strengths and limitations. While template-free techniques can be used for the mass production of low-cost but efficient plasmonic substrates, templated approaches offer an unprecedented synthetic control. Thus, a special emphasis is given to templated electrochemical lithographies, which can be used to synthesize complex metal architectures with defined dimensions and compositions in one, two and three dimensions. These techniques provide a spatial resolution down to the sub-10 nanometer range and are particularly successful at synthesizing well-defined metal nanoscale gaps that provide very large electric field enhancements, which are relevant for both fundamental and applied research in plasmonics.

## 1. Introduction

Nanostructured metals have highly tunable size- and shape-dependent optical properties. The excitation of localized surface plasmon resonances (LSPR) within metal nanostructures can largely enhance the incident electric field (E-field) in the near-field region, which strongly modifies light absorption and emission processes near the surface of the resonant structure [1,2,3,4,5,6,7]. This rich and complex interaction with light has been used for a variety of applications, ranging from surface-enhanced spectroscopies to photodetection, photovoltaics, and photocatalysis [8,9,10,11,12]. The strength and spatial distribution of the E-field and the range of energies at which it is the highest strongly depend on the size, shape, and three-dimensional order of the nanostructures [1,3,13,14,15,16]. So far, plasmonic nanostructures separated with nanoscale gaps have been the most efficient at achieving large field enhancements [14,15,17,18]. However, controlling such nanoscale gaps and integrating them within functional three-dimensional architecture is synthetically demanding.

To date, colloidal syntheses have been particularly successful at making metal nanoparticles (NPs) with uniform size, shape, and crystal structure [19,20,21,22]. As such, colloidal metal NPs have well-defined optical properties and have been the gold standard for plasmonics [4,13]. A number of self-assembly approaches have been developed to organize colloidal NPs into one, two, and three dimensions [16,23,24]. Although highly versatile, these methods can hardly be used to reliably introduce nanometer gaps between NPs, which are required to achieve strong plasmonic coupling. Until recently, electron beam lithography has been the method of choice for preparing such nanogaps, providing a ca. 2–5 nanometer resolution [7]. However, electron beam lithography is slow, not amenable to the production of large samples, requires expensive instrumentation, and is only compatible with the modification of surfaces (i.e., two-dimensional substrates).

Instead, electrochemical deposition (ECD) can provide a direct, simple, and cost-effective way to deposit metals on a conducting substrate. It is a powerful tool that can also be used to synthesize metal oxides, hydroxides, selenides, and other semiconductors that are relevant for a broad range of applications and are particularly useful for investigating, preparing, and developing new energy conversion and storage materials [25,26,27,28]. In addition to conventional ECD, a variety of electrochemical techniques have been developed to control metal nanostructure size, shape, porosity, and location in one, two, and three dimensions, with a spatial resolution that can go down to one nanometer. Herein, we discuss the electrochemical synthesis of metal nanostructures in the context of plasmonics. After a short introduction into plasmonics (Section 2), we review the recent advances made in using electrochemical approaches to synthesize metal nanostructures with well-defined dimensions and locations in one, two, and three dimensions (Section 3 and Section 4). The distinction is made between the template-free approaches (Section 3) and those requiring a templating structure (Section 4). Each synthetic method is presented in the context of its potential or reported use for plasmonics. In particular, we highlight three versatile electrochemical lithography techniques (Section 4.3 and Section 4.4) [29,30,31] to synthesize nanoscale metal gaps within complex systems that are shown to provide large E-field enhancements. Section 5 summarizes the advantages and limitations of the electrochemical syntheses presented in this review and provides an outlook on the use of electrochemical methods for nanostructuring plasmonic materials.

## 2. Optical Properties of Metal Nanostructures

Metals have a high density of free electrons that are able to oscillate under the incident electric field, similar to a gas of free electrons with a characteristic plasma frequency $\omega_{p}$, equal to

(1) $$\omega_{p}=\sqrt{\frac{ne^{2}}{m_{eff}\varepsilon_{0}}},$$

where *n* is the metal electron density, *e* the elementary charge, $m_{eff}$ is the effective electron mass, and $\varepsilon_{0}$ is the vacuum electric permittivity. The light–metal interaction is described by the frequency-dependent dielectric function of the metal, $\varepsilon_{metal}$, with $\varepsilon_{metal}=\varepsilon_{metal}^{\prime }+i\varepsilon_{metal}^{\prime \prime }$, where $\varepsilon_{metal}^{\prime }$ is the real part and $\varepsilon_{metal}^{\prime \prime }$ is the imaginary part of $\varepsilon_{metal}$, respectively. The plasma model can be used to express $\varepsilon_{metal}$ as a function of $\omega_{p}$ and the angular frequency, ω, as

(2) $$\varepsilon_{metal}\left(\omega \right)=1-\frac{\omega_{p}^{2}}{\omega^{2}+i\gamma \omega },$$

where γ is a characteristic collision frequency, typically around 100 THz. Thus, the metal optical properties depend on the value of ω relative to $\omega_{p}$: at $\omega >\omega_{p}$, the real part of the metal dielectric constant is positive and the metal behaves as a dielectric material, while at $\omega ≲\omega_{p}$, $\varepsilon_{metal}^{\prime }$ is negative and mostly real (negligible damping, $\varepsilon_{metal}^{\prime \prime }\sim 0$) and electromagnetic waves cannot propagate through the metal, leading to a reflection of the incoming photons.

### 2.1. Spherical Nanoparticles

**Figure 1:**
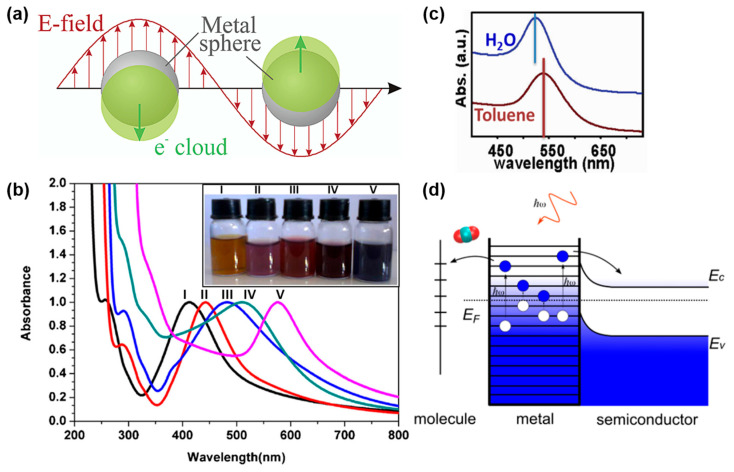
Localized surface plasmon resonance. (**a**) Schematic of the LSPR. (**b**) The effect of composition: UV−Vis spectra of AgAu alloy NPs with different compositions. I: Ag, II: Au_0.25_Ag_0.75_, III: Au_0.50_Ag_0.50_, IV: Au_0.75_Ag_0.25_, V: Au. (**c**) The effect of the surrounding medium: spherical Au NPs in water and in toluene. (**d**) Energy diagram showing the excitation of carriers with photons inside the metal and their transfer to a neighboring molecule or semiconductor. (**a**) Reprinted with permission from ref. [2]. Copyright 2003 American Chemical Society. (**b**) Reprinted from ref. [40], Copyright (2014), with permission from Elsevier. (**c**) Adapted with permission from ref. [41]. Copyright 2012 American Chemical Society. (**d**) Reprinted with permission from ref. [38]. Copyright 2016 American Chemical Society.

When metals are nanostructured into spherical NPs with sizes smaller than the wavelength of the incoming light, the uniform displacement of the conduction free electrons by the incident field that oscillates against the restoring force of the metal atom positive nuclei leads to an induced oscillating dipole, p, across the particle (Figure 1a) that can be expressed as:

(3) $$p=4\pi \varepsilon_{0}\varepsilon_{metal}R^{3}\frac{\varepsilon_{metal}-\varepsilon_{diel}}{\varepsilon_{metal}+2\varepsilon_{diel}}E_{0},$$

where R is the radius of the NP and $E_{0}$ the incident electric field amplitude. By introducing the polarizability, α, with $p=\varepsilon_{0}\varepsilon_{metal}\alpha E_{0}$, we arrive at the following expression

(4) $$\alpha =4\pi R^{3}\frac{\varepsilon_{metal}-\varepsilon_{diel}}{\varepsilon_{metal}+2\varepsilon_{diel}}.$$

The polarizability has a resonant enhancement at a frequency corresponding to the minimum of $\left|\varepsilon_{metal}+2\varepsilon_{diel}\right|$, which, when $\varepsilon_{metal}^{\prime \prime }$ is either low or varies slowly around the resonance frequency, simplifies to: [3]

(5) $$\varepsilon_{metal}^{\prime }=-2\varepsilon_{diel}.$$

The frequency at which the resonance occurs is the localized surface plasmon resonance (LSPR) frequency, $\omega_{LSPR}$, and is equal to

(6) $$\omega_{LSPR}=\frac{\omega_{p}}{\sqrt{1+2\varepsilon_{diel}}}.$$

At the LSPR, the metal NP strongly scatters and absorbs light (small NPs mostly absorb, while larger NPs mostly scatter light) [32], leading to their characteristic color, which depends on their composition (i.e., $\varepsilon_{metal}^{\prime }$) (Figure 1b) and the dielectric medium that surrounds them (i.e., $\varepsilon_{diel}$) (Figure 1c). Mie theory can be used to derive the exact solutions of the extinction, scattering, and absorption cross-sections in the case of a spherical metal NP [33]. The expression of the extinction cross-section, $\sigma_{ext}$, derived by Mie is

(7) $$\sigma_{ext}=9\left(\frac{\omega }{C}\right){\left(\varepsilon_{diel}\right)}^{3/2}V\frac{\varepsilon_{metal}^{\prime \prime }}{{\left(\varepsilon_{metal}^{\prime }+2\varepsilon_{diel}\right)}^{2}+{\left(\varepsilon_{metal}^{\prime \prime }\right)}^{2}},$$

where V is the volume of the NP. Thanks to their appropriate dielectric functions, the best plasmonic materials in the visible range are Ag, Au, and Cu [34]. At the LSPR, the metal NP concentrates the incoming far-field radiation at the surface of the NP by enhancing the near E-field due to the dipole induced in the metal NP (Figure 1a). Plasmonic excitations relax through both radiative (scattering) and non-radiative (hot carrier generation) processes (Figure 1d) [9,35,36,37,38,39]. Due to the quick thermalization of the hot electrons (100 fs timescale), the non-radiative decay channel can also lead to lattice heating.

### 2.2. Anisotropic Nanoparticles

**Figure 2:**
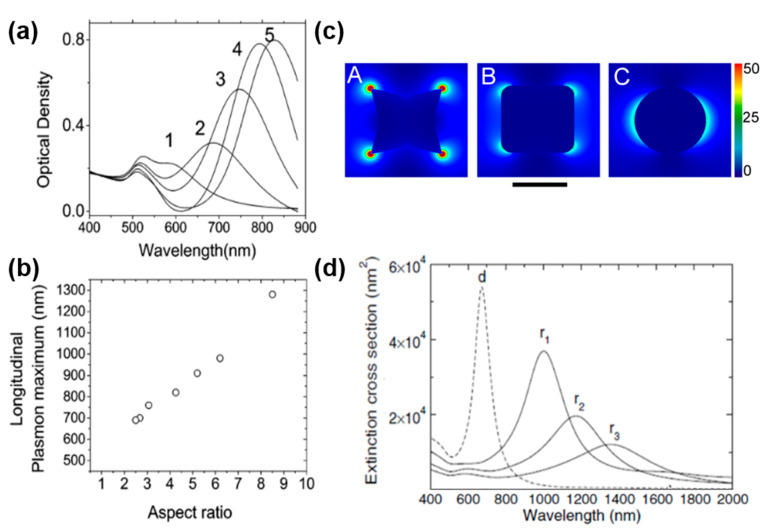
Anisotropic metal NPs. (**a**,**b**) Au NRs. (**a**) Visible-NIR spectra of five NR solutions with increasing aspect ratios, showing two distinct LSPR bands, one around 520 nm (transverse mode) and one at higher wavelengths (longitudinal mode). (**b**) Dependence of the longitudinal mode resonance wavelength as a function of the NR aspect ratio. (**c**) FDTD simulations of the near-field enhancements (E^2^) of Au NPs with different shapes plotted for a concave nanocube (A), a nanocube (B), and a nanosphere (C) in air with 785 nm excitation wavelength. The scale bar is 100 nm. (**d**) Experimental extinction spectra of a Au nanodisk (d, dashed line) and nanorings (r, solid lines) with an outer diameter of 120 nm and different wall thicknesses (decreasing from r_1_–r_3_). (**a**,**b**) Reprinted with permission from ref. [42]. Copyright 2003 American Chemical Society. (**c**) Reprinted with permission from ref. [45]. Copyright 2012 American Chemical Society. (**d**) Reprinted with permission from ref. [46]. Copyright (2003) by the American Physical Society.

Small spherical metal NPs can sustain one dipolar LSPR (the transverse and the longitudinal modes are degenerated due to the spherical symmetry). However, it is possible to engineer the plasmonic behavior of the metal NP either by increasing its size, in which case the NP can sustain higher order multipolar resonances, or by engineering the NP shape to produce an anisotropic NP. Such anisotropic NPs can sustain different LSPRs along specific directions due to the anisotropy of the polarizability. For example, metal nanorods (NRs) have a transverse mode at high frequencies and a longitudinal mode at lower frequencies. By adjusting the aspect ratio of the metal NRs, it is possible to tune the longitudinal mode resonance frequency and, therefore, the energies at which the E-field will be enhanced (Figure 2a,b) [13,42]. This can be used to control the surface E-field enhancement at the end of metal NRs and the light absorption as a function of wavelength [43,44]. Interestingly, while the highest surface E-fields are observed at the ends of the plasmonic NR, electromagnetic simulations indicate that the highest bulk E-field and, thus, light absorption occurs in the middle of the NR [44].

Since the largest E-field enhancements at plasmonic NPs are obtained at the regions with the highest curvature [2], even small differences in the shape of NPs can have large effects on their plasmonic properties. For example, it was demonstrated that concave nanocubes exhibit significantly larger SERS enhancement factors (e.g., 60 times larger) compared to regular nanocubes and even larger enhancements compared to spherical Au NPs [45]. This was underlined by FDTD simulations (Figure 2c), which demonstrate that the highest E-field enhancement is obtained at the edges of the concave Au nanocubes.

Metal nanorings are another interesting type of anisotropic nanostructure because of their unique electromagnetic properties: they can support three different LSPRs along the three possible electric field polarizations, and they can focus the incident light inside as well as outside the cavity. By tuning the ring inner and outer diameters, it is possible to fully tune the in-plane symmetric dipolar LSPR from the visible to the infrared range (Figure 2d) [46]. While the high tunability of the ring LSPRs is interesting from a plasmonic standpoint, the ability to enhance the incident electric field inside the ring cavity offers the opportunity to have a strong homogeneous optical coupling over large volumes. This has been used to develop highly sensitive molecular plasmonic sensors, thanks to the large amount of molecules that can interact with the enhanced field inside the ring cavity [47].

### 2.3. Controlling Distances within Metal Nanostructures in One, Two, and Three Dimensions

#### 2.3.1. Nanoscale Gaps (1D)

**Figure 3:**
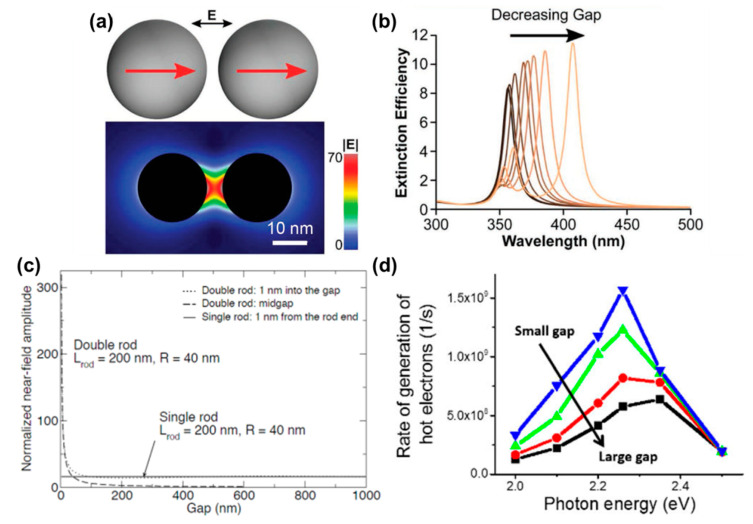
Enhanced E-field at metal nanogaps. (**a**,**b**) Ag NP dimer. (**a**) Schematic (top) and simulated E-field contour map (bottom) showing the large E-field enhancement in the gap region. (**b**) Simulated extinction efficiency as a function of gap size, showing the characteristic red shift of the LSPR with decreasing gap length due to the increased coupling between the two induced dipoles. (**c**) Near-field amplitude of a pair of identical Au NRs, coupled end-to-end, as a function of gap separation. The NRs were irradiated with a plane wave with longitudinal polarization. Corresponding results for an isolated rod are also indicated. (**d**) Simulated rate of hot electron generation within Au NP dimers (3 nm radius), going from a 1 nm gap to an infinite gap (blue curve to the black curve). (**a**,**b**) Reprinted from ref. [16]. Copyright 2016 American Chemical Society. Further permission related to the material excerpted should be directed to the ACS. (**c**) Reprinted from ref. [4]. Copyright 2008 WILEY-VCH (**d**) Reprinted with permission from ref. [38]. Copyright 2016 American Chemical Society.

Metal nanostructures separated by nanoscale gaps can lead to hot spots in the gap region where the E-field can be enhanced by an additional factor of 10 or even 100 at the resonance wavelength compared to the isolated structure (Figure 3a) [14,15,17,18], leading to their extensive use in surface-enhanced spectroscopy (fluorescence and Raman) [14,18,48]. In such cases, the induced dipolar near field of the neighboring NPs couple with each other, leading to a strong field localization in the gap as well as a red shift in the LSPR. Under longitudinal polarization, coupled NP dimers act as an anisotropic rod-like structure rather than a collection of two isolated spheres (Figure 3b). In general, the smaller the gap the larger the E-field enhancement at the resonance (Figure 3c). Additionally, recent quantum mechanical simulations suggest that such gaps can significantly enhance hot electron generation (Figure 3d) [38]. Thus, controlling nanoscale distances between well-defined metal NPs is crucial to optimize the E-field enhancement.

#### 2.3.2. One-, Two-, and Three-Dimensional Plasmonic Arrays (1D, 2D, and 3D)

In addition to using metal nanogaps to achieve strong plasmonic coupling, ordering plasmonic NPs within one-, two- and three-dimensional arrays has emerged as a versatile approach to tune a material’s plasmonic response [16]. Schatz et al. originally investigated the generation of sharp LSPRs within periodic 1D and 2D arrays of silver NPs separated by distances commensurate with the LSPR wavelength [49,50]. Diffractive coupling of the light scattered by the metal particles leads to so-called lattice plasmon modes, which have a characteristic narrow line width (down to ca. 1 meV). Such lattice resonances are strongly dependent on the array periodicity, can be tuned through a wide range of wavelengths, and enhance the particle near field [49,50,51]. Three-dimensional plasmonic arrays provide an additional means to tune the material’s refractive index, have unconventional wavelength-dependent transmission and reflective properties, and provide a great platform to couple the plasmonic and photonic resonances [16]. While 1D and 2D arrays can be prepared via a variety of methods, the synthesis of 3D plasmonic crystals is quite challenging, usually requiring the use of DNA-based self-assembly [16].

## 3. Template-Free Electrochemical Synthesis of Metal Nanostructures

### 3.1. Electrochemical Deposition

Because most transition metal ions can be reduced in aqueous solution, electrochemical deposition (ECD) is an effective way of coating conducting substrates with conformal metal films [25]. Due to its low cost and simplicity, ECD is routinely used in industry. For example, ECD has been instrumental in the miniaturization of microprocessors: In 1997, IBM replaced Al metallic interconnects for microchips with Cu due to the higher electrical conductivity of Cu. Since Cu was not compatible with the physical vapor deposition/reactive ion etching steps previously developed to process Al, it was necessary to develop a new way of micropatterning Cu. The so-called damascene process developed by IBM relies on the electrodeposition of Cu within photolithographically defined tracks on the surfaces of the chips [52,53]. It has considerably contributed to promoting the manufacture of faster and smaller microprocessors.

#### 3.1.1. Synthesis of Monodisperse NPs

Aside from being well-suited for industry, ECD is a well-established and versatile method to prepare conformal and nanostructured coatings for electrochemical research [25]. However, the electrochemical synthesis of monodisperse metal NPs is not as straightforward as one might think: as detailed by Penner, conventional potentiostatic ECD leads to polydisperse NP distributions [54]. This is partly due to the continuous nucleation of NPs at the substrate surface, which occurs during NP growth. To obtain monodisperse NP samples via solution-phase synthesis, LaMer suggested 70 years ago to separate in time NP nucleation from NP growth [55]. This avoids the formation of new small nuclei over time, which would otherwise grow to smaller sizes than the NPs formed in the early stages of the reaction. Additionally, NP growth should be diffusion-controlled rather than kinetically controlled, as suggested by Reiss, to allow the smaller NPs to grow faster than the larger ones, with the direct effect of narrowing the particle size distribution [56]. This becomes evident when we look at the change in the particle radius, R, as a function of time under conditions of kinetic control [57],

(8) $$\frac{dR}{dt}=kV_{m}C^{*}$$

where k is the surface reaction rate, which is assumed to be independent of particle size, $V_{m}$ is the molar volume, and $C^{*}$ is the bulk concentration, and under diffusion control:

(9) $$\frac{dR}{dt}=\frac{DV_{m}C^{*}}{r}$$

Equations (8) and (9) assume that the NP has negligible solubility in solution, which is quite accurate for noble metals. The particle growth rate under kinetic control is constant over time, thus polydisperse NP samples preserve the same level of size dispersity over time. On the other hand, diffusion-controlled growth leads to a slower growth of larger particles and, therefore, to a narrowing of the NP dispersity over time. This strategy has been used for a long time to prepare monodisperse NP samples via conventional solution-phase colloidal syntheses, which can explain the overwhelming number of successful NP syntheses based on conventional colloidal chemistry [57,58,59] compared to electrochemical approaches, with the exception of the first reported (and highly cited) template-free synthesis of Au NRs that was based on ECD [60].

**Figure 4:**
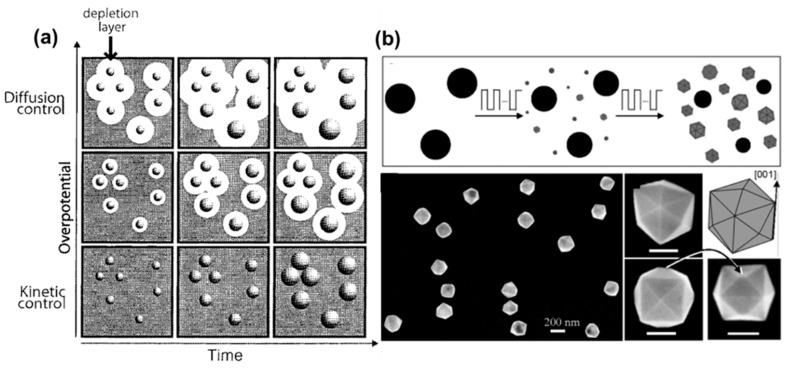
Electrochemical synthesis of metal NPs. (**a**) Schematic showing the effect of overpotential on the reaction rate and the size dispersity of metal NPs synthesized via electrochemical deposition. (**b**) Top: Scheme of electrochemical preparation of tetrahexahedral Pt NPs from spherical NPs. By applying a squarewave potential, new Pt NPs grow at the expense of the large spherical NPs that dissolve, while the newly nucleated NPs eventually transform into tetrahexahedral shapes. Bottom: typical SEM images of the Pt NPs. Scale bars: 100 nm. (**a**) Reprinted with permission from ref. [54]. Copyright 2002 American Chemical Society. (**b**) From [61]. Reprinted with permission from AAAS.

The situation is quite different during the electrochemical deposition on a conducting electrode, where NPs can nucleate close from each other, leading to the so-called “interparticle diffusion coupling”, where the depletion layer around nearby NPs merge, leading to a decrease in the reactant concentration and, therefore, in the growth rate. Because of the random NP nucleation at the electrode surface, not all NP depletion layers overlap with the same number of NPs, leading to an inhomogeneous growth rate at the electrode surface. This explains the much larger NP size distribution that is obtained via electrodeposition compared to traditional colloidal syntheses [54]. Penner and coworkers reported an elegant way to mitigate this problem by separating the nucleation from the growth using a double plating pulse technique: an initial large overpotential is used during a short period of time for the heterogeneous nucleation, while a second longer pulse at a lower overpotential insures a slow uniform growth while preventing further nucleation (Figure 4a). This slow growth prevents the depletion layers from neighboring particles from overlapping with each other, leading to a uniform kinetically controlled NP growth across the electrode that improves the NP size dispersity.

#### 3.1.2. Synthesis of Anisotropic NPs

Since the work from Penner, ECD has been used to synthesize facetted metal NPs with well-defined crystallographic orientations, such as Pt NPs with tetrahexahedral shapes that are enclosed by 24 high-index facets and have a large density of atomic steps and dangling bonds [61]. The NPs were synthesized via a multistep electrochemical process based on the cyclic electrochemical oxidation/dissolution and reduction of electrodeposited Pt NPs [61]. Since then, others have successfully followed similar strategies [62,63,64]. Remarkably, electrochemistry can be used to improve our current understanding of colloidal syntheses by measuring the time evolution of some key electrochemical parameters during synthesis. For example, it is possible to measure the time evolution of the electrode open circuit potential (OCP) in the presence of a specific reducing agent. Because kinetics play an important role in the growth of metal NPs obtained by reducing metal ions in solution [22], reducing agents with different standard potentials can yield NPs of different shapes. By programming the working electrode potential to reproduce some of the OCP time evolution, it is possible to electrochemically reproduce the conditions of specific colloidal syntheses and electrosynthesize complex anisotropic metal NPs [64]. As such, electrochemistry can be used for both synthesizing anisotropic metal NPs and potentially achieving complex morphologies that could not otherwise be obtained via conventional wet-chemical approaches [64].

Potential plasmonic applications:

The electrochemical synthesis of anisotropic NPs with sharp features and complex morphologies, known to significantly enhance the near E-field, is especially interesting for plasmonics. Because such NPs offer surfaces with a large number of atomic steps and dangling bonds, they are well-suited for (electro)catalysis, as demonstrated, for example, by the high electrocatalytic activity of tetrahexahedral Pt NPs for the electro-oxidation of formic acid and ethanol [61]. Recently, colloidal Rh nanocubes have shown a high selectivity towards the methanation of CO_2_ under UV/blue illumination [65]. Thus, high-index facet Rh NPs, such as the tetrahexahedral Rh NPs synthesized via an electrochemical square wave potential method [66], could potentially further enhance reaction rates and selectivity by providing a large number of sharp edges and dangling bonds with plasmonic photocatalytic activity.

### 3.2. Ostwald Electrochemical Ripening

**Figure 5:**
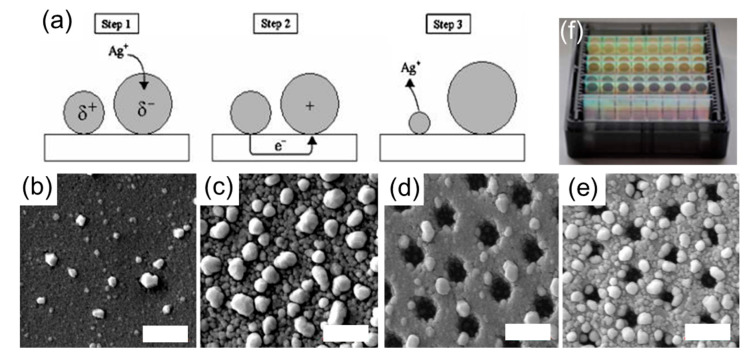
Electrochemical Ostwald ripening. (**a**) Schematic showing the reduction of Ag^+^ on the large NPs followed by the electron transfer from the small to the large NPs and the oxidation of Ag(0) NP into Ag^+^. (**b**–**e**) SEM images showing the morphology of a sputtered Ag film on polymeric substrates with flat surfaces (**b**,**c**) and microstructured surfaces (**d**,**e**) after being incubated in an acidic aqueous solution for 1 min (**b**,**d**) and 100 min (**c**,**e**). (**b**–**e**) Scale bars are 1 micron. (**f**) Photograph of SERS substrates prepared via injection molding and Ostwald electrochemical ripening of a sputtered Ag film, leading to a nanostructured Ag surface such as the one shown in (**e**). (**a**) Reprinted with permission from ref. [67]. Copyright 2005 American Chemical Society. (**b**–**f**) Reprinted with permission from ref. [70] Copyright 2017 American Chemical Society.

In 2005, Brus et al. reported the spontaneous dissolution/recrystallization of nanostructured Ag films on conducting substrates (indium tin oxide—ITO and highly ordered pyrolytic graphite—HOPG) that were exposed to aqueous solutions [67]. With increasing incubation times, the larger NPs grow at the expense of the smaller NPs that dissolve (Figure 5). The room temperature reduction of bulk Ag is described by the half-cell reaction (Equation (10)), with a standard electrode potential of $E_{bulk}^{0}=+0.80\;V\;\mathrm{vs}.$ the standard hydrogen electrode (SHE).

(10) $${\mathrm{Ag}}_{\left(\mathrm{aq}\right)}^{+}+e^{-}\to {\mathrm{Ag}}_{\left(s\right)}$$

However, the standard electrode potential of a metal NP shifts negatively as the NP becomes smaller. This is described by the size-dependent electrode potential, $E_{NP}^{0}\left(R\right)$, derived by Plieth et al. (Equation (11)), where $\gamma_{s}$ is the surface tension, z is the lowest valence state, F is the Faraday constant, and R is the radius [67,68,69].

(11) $$E_{NP}^{0}\left(R\right)=\left(E_{bulk}^{0}-\frac{2\gamma_{s}V_{m}}{zFR}\right)$$

Due to the 1/R dependency, the shift is noticeable, even for quite large NPs (R < 100 nm), while being significant for NPs with R < 5 nm. Ag NPs have a finite solubility that is dictated by the dissolved O_2_ concentration according to Equation (12):

(12) $$4\mathrm{Ag}+O_{2}+4H^{+}\to 4{\mathrm{Ag}}^{+}+2H_{2}O$$

Thus, smaller NPs tend to dissolve more and have a higher concentration of Ag^+^ ions around them. Because small and large NPs have different $E_{NP}^{0}$, they have different work functions. When immersed into an electrolyte and at equilibrium, they develop a different partial electric charge. This electric field attracts the Ag^+^ ions generated at the small positively charged NPs to the larger negatively charged Ag NPs where they are reduced (Figure 5a).

These electrochemical processes lead to an electrochemical Ostwald ripening, where the large NPs grow bigger and the small NPs dissolve (Figure 5b,c). As expected, electrochemical Ostwald ripening does not occur if the substrate is biased with a reducing potential or if a protecting ligand, such as sodium citrate, is added to the electrolyte. Additionally, it is accelerated when additional Ag^+^ ions are added to the solution or when acidic solutions are used. Such a reforming phenomenon has also been observed under specific conditions when the substrate is electrically insulating [70,71], in which case the Ag film itself acts as the conducting electrode.

Plasmonic applications:

The etching of sputtered Ag films on microstructured polymeric substrates (Figure 5d,e) yields a highly dense Ag NP film at the sample surface, which provides a very large and uniform enhancement of the electric field, as demonstrated by Raman spectroscopy measurements [70]. Since these microstructured samples can be mass-produced via injection-molding, the use of such an electrochemical Ostwlad ripening provides a way of mass-producing SERS substrates with high sensitivity and high signal reproducibility that can be sold for less than USD 1 a piece [70]. Additionally, the electrochemical Ostwald ripening of Ag micro-hemispheres was used to synthesize parallel Ag nanoplates with controllable gap sizes down to 4 nm, which significantly enhanced the SERS signal compared to the unetched samples [72]. These experiments demonstrate the overall instability of nanostructured Ag in an electrolyte, while providing an elegant, cost-effective, and electroless means of controlling the morphology of nanostructured Ag films, which can then be used outside the electrolyte as efficient substrates for SERS [70,72] and surface-enhanced fluorescence [72,73].

### 3.3. Electrochemical Dealloying

Instead of reducing metal ions on an electrode, the selective electrochemical oxidation of metal alloys provides a simple way to nanostructure metals, which can be suitable for plasmonic applications. Such an electrochemical dealloying is based on the differences in the standard potentials of different metals, which allows for the selective dissolution of one of the metal components [74]. The metal alloy can be prepared via different techniques, such as, for example, physical vapor deposition [75,76] or electrodeposition [77]. By immersing the alloy into an electrolyte and applying a potential to oxidize the less noble metal only, it is possible to selectively dissolve one metal component, which is referred to as dealloying. This critical potential, V_C,_ depends on the composition of the alloy and increases with the increasing fraction of the more noble metal [74,78]. Despite starting from an alloy with a homogenous metal distribution, the electrochemical dealloying leads to the formation of nanoporous three-dimensional networks with open pores. This is because of the non-uniform atomic distribution that arises during the dealloying process. The dealloying rate-limiting step is the dissolution of the less noble metal atoms from terrace sites. This leads to the diffusion of the neighboring more noble metal atoms—which are under-coordinated—that passivate the resulting surface. With continuing dealloying, the more noble metal cannot totally passivate the increasing surface area, yielding a bicontinous structure composed of sub-50 nm metal filaments. The surface and bulk of these filaments are rich in the more noble metal and the less noble metal component, respectively. Since nanostructured metals are less stable than the bulk, the typical length scales of the dealloyed structure can be increased, e.g., by a heat treatment, and such filaments can then be grown up to several microns in length [74]. Similarly to the electrochemical Ostwald ripening, electrochemical dealloying allows the formation of nanostructures separated with gaps as small as a few nanometers, although the gap size distribution is rather large. While dealloying can also be performed via selective wet-chemical etching, electrochemical dealloying provides a better control over the dealloying kinetics, which is crucial to synthesizing ultralow-density nanoporous gold (ULDNPG) from AuAg alloys [76].

**Figure 6:**
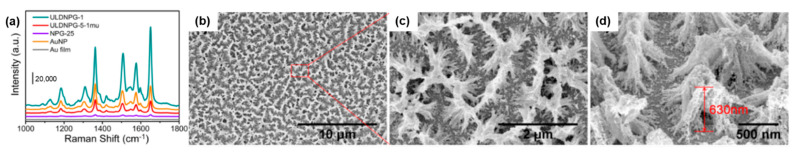
Electrochemical dealloying. (**a**) SERS spectra of rhodamine 6G (concentration is 1.0 × 10^−6^ M) obtained from ULDNPG−1 (dealloyed Au_1_Ag_99_ alloy thin film with 1 µm thickness), ULDNPG−5−1mu (dealloyed Au_5_Ag_95_ alloy thin film with 1 µm thickness), AuNP array, NPG−25, and Au film, respectively. (**b**–**d**) SEM images of ULDNPG−1 prepared via electrochemical dealloying. (**a**–**d**) Reprinted with permission from ref. [76]. Copyright 2021 American Chemical Society.

Plasmonic applications:

ULDNPG synthesized via electrochemically dealloying AuAg alloys (1% Au molar fraction) is composed of many highly curved features that provide higher E-field enhancement and, thus, SERS activity than isolated Au NPs (Figure 6) [76]. Such ULDNPG films have also shown better photocatalytic properties than Au NPs [76].

## 4. Templated Electrochemical Syntheses

Instead of relying on electrochemical oxidation and/or reduction on planar substrates, the use of well-defined sacrificial nanostructures, patterned planar substrates, and porous membranes has emerged as a powerful way to control metal nanoscale architecture.

### 4.1. Sacrificial Templates: Electrochemical Reduction of Non-Zero-Valent Metal Nanostructures

**Figure 7:**
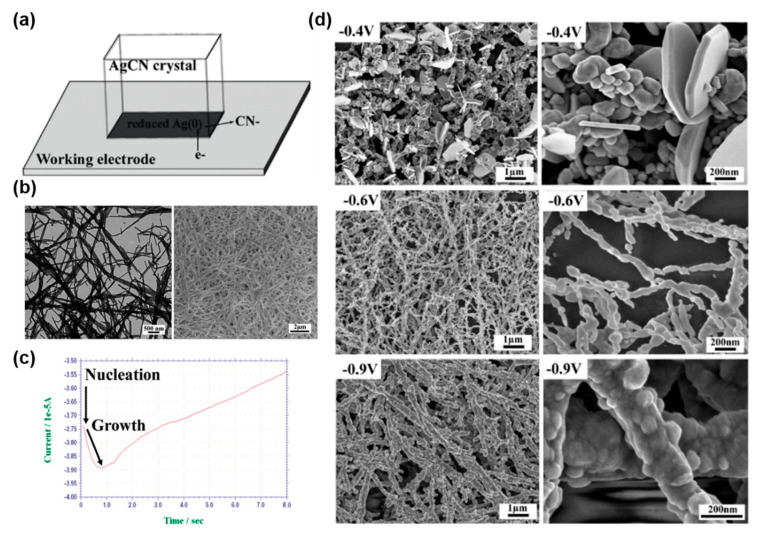
Electrochemical reduction of non−zero−valent metal nanostructures. (**a**) Schematic of the electrochemical reduction of solid AgCN. (**b**) TEM (left) and SEM (right) images of AgCN nanofibers. (**c**) Current measurement during the early stages of the electrochemical reduction of an NZVN on a graphite electrode (−0.55 V for 1200 s). (**d**) SEM images of Ag^0^ nanostructures with different morphologies formed on the glass substrate via the reduction of AgCN nanofibers as a function of the reducing potential in 0.1 M KClO_4_. (**a**–**d**) Reprinted with permission from ref. [80]. Copyright 2010 American Chemical Society.

An alternative to the reduction of metal ions to form metal NPs is the reduction of insoluble nanostructured metal complexes. A variety of anisotropic non-zero-valent metal nanostructures (NZVN) can be synthesized via self-assembly and colloidal synthesis [79,80,81,82,83]. Such NZVN can be used as sacrificial templates, yielding well-defined metallic nanostructures after reduction [79,80,81,82,83]. In general, the resulting structure geometry depends on the parent template shape and dimensions and the reduction kinetics: a fast reduction tends to preserve the NZVN morphology as much as the change in volume accompanying the conversion allows it. For example, the fast chemical reduction of Au(I)Cl(*n*-butylamine) nanofiber sheets leads to nanoporous monoliths composed of nanostructured Au(0) fibers [79]. However, a slower reduction can favor more thermodynamically stable products, leading to significant geometrical changes. One of the great advantages of electrochemistry is the possibility to tune the Fermi level of the working electrode and, thus, to adjust reduction kinetics, allowing for a more precise control of the final product morphology. Indeed, by controlling the potential during the electrochemical reduction of AgCN nanofibers, it is possible to obtain different Ag(0) morphologies [79,80]. At highly reducing potentials, i.e., −0.6 V to −0.9 V vs. Ag/AgCl, the Ag(I) nanofiber morphology is preserved (Figure 7). However, at moderately reducing potentials, i.e., around −0.4 V vs. Ag/AgCl, the reduction is slower, and the Ag(I) fibers are converted into one-dimensional assemblies of large Ag NPs and Ag nanoprisms, whose synthesis depend on crystallographic and kinetic factors [19,84]. Similarly, Cu(OH)_2_ nanowires, which spontaneously form in aqueous solution, can be electrochemically reduced into Cu(0) nanowires [80] and can be later converted into nanoporous platinum via a subsequent galvanic exchange [85].

Potential plasmonic applications:

The electrochemical reduction of NZVN can be used to synthesize a variety of large (i.e., cm^2^-scale) metal nanostructured networks, which provide a rough and three-dimensional morphology and a high density of nanoscale metal gaps (Figure 7d) that are well-suited for SERS and plasmonic catalysis.

### 4.2. Patterned Planar Electrodes

**Figure 8:**
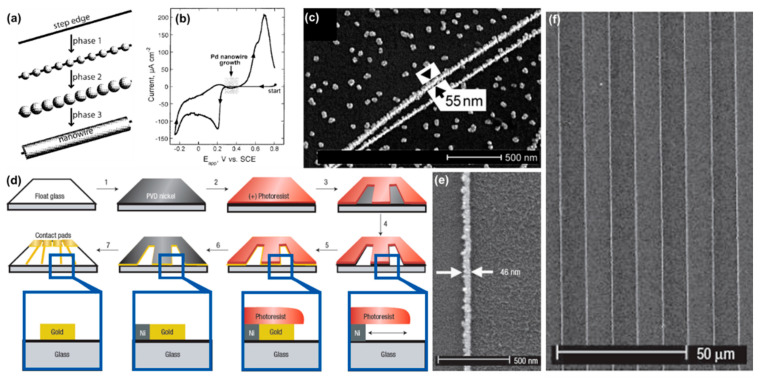
Electrochemical deposition on patterned planar electrodes. (**a**–**c**) Step edge decoration. (**a**) Schematic description of the three phases. (**b**) Cyclic voltammogram of a graphite electrode in aqueous Pd solution (2.0 mM Pd^2+^, 0.10 M HCl). The potential range used for the growth of Pd nanowires is marked in gray. (**c**) SEM micrograph of Pd nanowires prepared by electrodeposition from aqueous solution. (**d**–**f**) Lithographically patterned nanowire electrodeposition (LPNE). (**d**) Schematic of the seven steps involved. (**e**) SEM image of a Pt nanowire with a width of 46 nm and a length of more than 1 cm. (**f**) SEM image of parallel Au nanowires. (**a**,**b**) Reprinted with permission from ref. [54]. Copyright 2002 American Chemical Society. (**c**) From [87]. Reprinted with permission from AAAS. Copyright 2001 American Association for the Advancement of Science. (**d**–**f**) Reprinted by permission from ref. [88], Copyright 2006 Nature.

Substrates such as single crystals and HOPG are composed of atomically flat terraces and step edges. Because of their lower coordination number, heterogeneous nucleation is more likely to occur at the atoms located at the step edges. This has been used to synthesize nanowires, which are formed along step edges via various physical vapor deposition methods [86] as well as electrochemically [87]. Similar to the electrochemical deposition of metal NPs on flat substrates, a two-step pulsed method can be used to initiate nucleation at the step edges with a high overpotential, which is, however, lower than the one used for NP nucleation at the terraces, and a second longer pulse with a much lower overpotential for achieving a controlled slow growth [54]. As shown in Figure 8a–c, after nucleating at the step edges, the NPs grow until they coalesce with their nearest neighbors, forming a “beaded” nanowire. Further growth leads to a smoothing of the nanowires.

While electrochemical deposition at step edges offers the advantage of simplicity, it is limited by the fact that the size, density, and orientation of step edges at the HOPG surface cannot be controlled externally. As such, this approach cannot be used to control the distance separating the nanowires. To remediate this problem, the Penner group developed the lithographically patterned nanowire electrodeposition (LPNE) technique [88]. LPNE is based on well-defined Ni nanobands that are patterned on an insulating substrate via a combination of photolithography and electrochemical oxidation and is used as a working electrode to plate the target metal. Importantly, during the electrode preparation, a trench is generated below the photoresist, forming a horizontal rectangular opened track that gives access to the electrolyte for the subsequent ECD of the target metal nanowires. The dissolution of the Ni electrode yields metal nanowires at the substrate surface. Figure 8d shows a schematic description of the fabrication process. The produced nanowires have a rectangular cross-section whose height is defined by the nickel film thickness (adjustable with a 5 nm precision down to 18 nm), and width is adjusted by the ECD step (adjustable down to 40 nm). The advantage of the technique is its simplicity and the ability to grow a variety of metal and semiconductor nanowires on planar substrates using a combination of photolithography and simple electrochemical techniques. A modified version of the LPNE, which is based on nanosphere lithography, was used to synthesize metal nanoring arrays with tunable diameters and pitches. By exchanging the plating solution, sequential ECD yields concentric heterometallic nanorings. By selectively etching Ni, concentric AuNiAu nanorings could be converted into concentric Au nanorings separated by sub-50 nm gaps [89].

**Figure 9:**
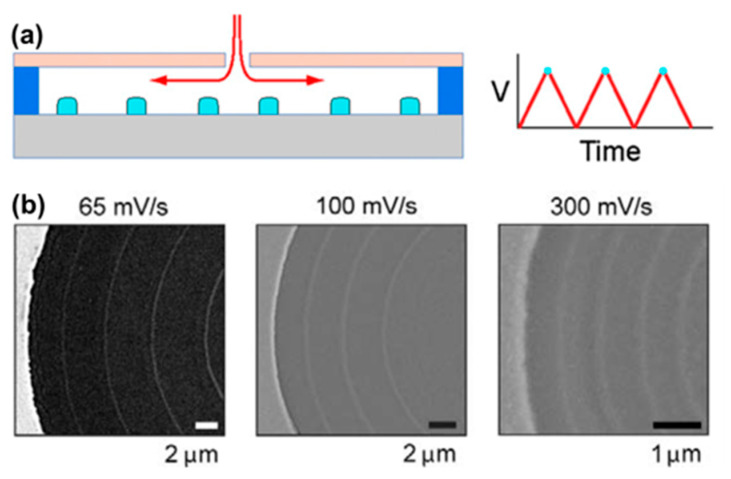
RIPPLE. (**a**) Schematic of RIPPLE. During linear sweeps, lateral etching of the parent material occurs in concert with site-specific localization of nanoscale periodic features (light blue). The pitch is controlled by the voltage scan rate. (**b**) SEM images of periodic Ge concentric rings patterned at different scan rates. Reprinted from ref. [90]. Copyright 2015 National Academy of Sciences.

Instead of growing metal nanowires on a nanostructured Ni template, the group of Nocera reported an intriguing electrochemical approach to prepare periodically spaced metallic and oxidic nanostructures, termed RIPPLE [90]. During RIPPLE, the conducting electrode is coated with a target material that is patterned with small micron-sized photoresist openings to limit the initial access of the solution to the electrode. Linear potential sweeps dissolve and redeposit the target material, leading to periodically spaced nanostructures below the photoresist, as shown in Figure 9. The width and height of the periodic nanostructures fabricated via RIPPLE are in the sub-100 nm range and, thus, should be able to sustain localized surface plasmon resonances, while the pitch is in the micrometer range, which is compatible with far-field coupling.

Plasmonic applications:

Both LPNE and RIPPLE allow the fabrication of nanostructures with adjustable distances between the nanowires and nanorings, which has implications for plasmonics. The modified LPNE technique used to prepare Au nanoring arrays with tunable dimensions and NIR absorption is especially interesting [89]. It provides the possibility to synthesize 2D arrays of concentric heterometallic nanorings with tunable dimensions and gap lengths, which are well-suited for studying plasmonic coupling within complex ring arrays without requiring expensive fabrication techniques, such as e-beam lithography.

### 4.3. Porous Membranes

#### 4.3.1. Nanorod Synthesis

**Figure 10:**
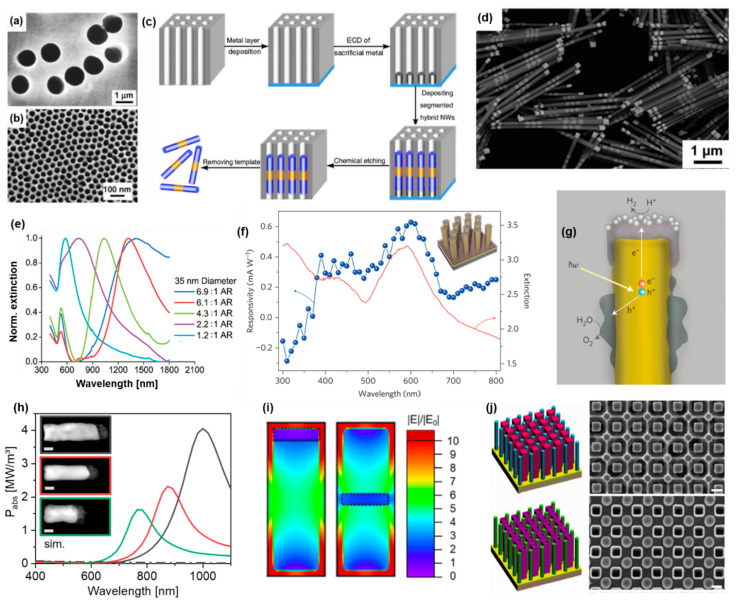
Electrochemical synthesis templated within porous membranes. (**a**,**b**) Top-view SEM images of an ion-track-etched membrane (**a**) and a porous alumina membrane (**b**). (**c**) Scheme of the synthesis of one-dimensional nanostructures by the electrodeposition of materials into nanoporous templates. (**d**) SEM image of multisegmented Au–Ni–Ag nanowires. (**e**) UV-Vis spectra of Au NRs with different aspect ratios synthesized in AAO membranes with 35 nm nominal pore diameters. (**f**) Photocurrent and extinction spectra of the photocathode (shown in the inset: Au NR arrays capped with TiO_2_ and decorated with Pt NPs). (**g**) Scheme of of an individual photocatalytic water-splitting unit (Au NR with a TiO_2_ cap decorated with platinum NPs, which functions as the hydrogen evolution catalyst, and the Co-based oxygen evolution catalyst deposited on the lower portion of the Au NR). (**h**) Simulated absorbed power density (longitudinal polarization of the incident E-field) within the Rh/Ru segment at the end of Au NRs with different Au segment lengths of the structures shown in the inset (green: AuRu; red: AuRu; black: AuRh). Scale bars are 20 nm. (**i**) Simulated E-field enhancement maps of a AuRh NR (left) and a AuRhAu NR (right) at longitudinal polarization at 818 nm excitation wavelength. The NRs have diameters of 40 nm and total lengths of 120 nm, and the length of the Rh segments is set to 10 nm. (**j**) Representative binary nanostructure arrays obtained using binary pore AAO templates; left: illustrations and right: corresponding top-view SEM images of top: nanowire/nanowire; bottom: nanowire/nanotube. (**a**,**b**) Reprinted from ref. [94]. Copyright 2006 Wiley-VCH. (**c**) Reprinted from ref. [104] Copyright 2018, with permission from Elsevier. (**d**) Reprinted with permission from ref. [24]. Copyright 2011 American Chemical Society. (**e**) Reprinted with permission from ref. [99]. Copyright 2010 American Chemical Society. (**f**,**g**) Reprinted by permission from ref. [102], Copyright 2013 Springer Nature. (**h**,**i**) Reprinted from ref. [44] Copyright 2021 American Chemical Society. Further permission related to the material excerpted should be directed to the ACS. (**j**) Reprinted by permission from ref. [98]. Copyright 2017 Springer Nature.

To synthesize NRs with well-defined diameters and lengths, the templated electrodeposition within the tubular pores of a porous membrane has emerged as a simple and versatile technique. The approach, originally pioneered by Moskovits [91] and Penner and Martin [92,93], has since been used to synthesize a wide range of nanowire-based devices, such as inorganic and organic-hybrid diodes, transistors, SERS substrates, micromotors, sensors, photoelectrodes, and plasmonic photocatalytic systems [94]. Commonly, anodic aluminum oxide (AAO) membranes or ion-track-etched membranes are used for this purpose (Figure 10a,b). Conducting wires are grown from the bottom to the top of the pores by coating one side of the membrane with a conducting metal layer, which is used as the working electrode. By controlling the charge consumed during the electrochemical process, it is possible to control the segment length from a few nanometers up to tens of micrometers, with a typical variation in segment length of ca. 10%. Multisegmented nanowires composed of different materials can be grown by sequentially changing the plating solution (Figure 10c). In addition to adjusting the NR composition and geometry, it is also possible to control the material’s crystallinity. Indeed, using appropriate deposition potentials, temperatures, and additives in the plating solution, single crystalline metal NRs can be grown for a variety of metals. In general, higher overpotentials lead to the formation of polycrystalline NRs [95]. Similarly, an increase in the overpotential and a decrease in the surfactant concentration lead to the formation of rather coarse Rh NRs [96]. Mallouk et al. reported that the ECD of NRs comprised of group VIII-B metals yields polycrystalline structures with small grain sizes (e.g., 2–5 nm for Rh) and large porosities. The ECD of these metals appears to be relatively insensitive to overpotential and the addition of additives [95]. Our group recently characterized the morphology and porosity of electrodeposited Rh and Ru NRs and estimated a porosity of ~40% for these metals, with grain sizes around 2 nm. Interestingly, despite their large porosity, the thin porous Rh and Ru segments (e.g., 10 nm thickness) could effectively bridge the plasmonic oscillations of Au NR dimers [44]. Due to their high surface area and ability to couple with plasmonic absorbers, such porous NR segments have potential for plasmon-enhanced photocatalysis.

By selectively etching the backing layer at the bottom of the membrane and dissolving the membrane, the nanowires can be dispersed in solution. Alternatively it is possible to dissolve the AAO membrane without etching the backing layer first to obtain freestanding nanowires [97]. A wide range of metals, alloys, and organic and inorganic semiconductors can be grown electrochemically, making templated electrochemical deposition a highly versatile technique [94]. It is also possible to engineer the AAO membrane to obtain binary arrays of pores with different cross-sections (rectangular or cylindrical, for example), as shown in Figure 10g, which can then be selectively used to electrochemically deposit different materials [98]. This is interesting from a plasmonic point of view because it allows the coupling of various arrays of one-dimensional nanostructures with different compositions, shapes, and dimensions.

Plasmonic applications:

The use of membranes with pore sizes in the sub-100 nm range provides the opportunity to synthesize metal NRs with well-defined LSPRs in the visible range [43,99]. By varying the length of the metal segments, it is possible to adjust the longitudinal LSPR wavelength (Figure 10e) [99]. This has been used to gain a deeper understanding of the influence of NR diameter and aspect ratio on the longitudinal LSPR wavelength: An equation predicting the longitudinal LSPR wavelength was derived for NR dimensions that are beyond the quasi-static limit [99]. Additionally, by synthesizing a light-emitting conjugated polymer segment at the end of a Au NR, it is possible to modulate light emission by controlling the overlap between the LSPR and the emission band of the polymer [43]. This was demonstrated by synthesizing Au–polythiophene (AuPTh) NRs showing strong emission in the 600–700 nm range: by varying the Au segment length, the longitudinal LSPR wavelength can be adjusted through the visible to the near-IR range [43].

Thanks to its high material generality and spatial resolution, templated electrochemical synthesis is the method of choice for studying plasmonic coupling within heterometallic nanostructures. For example, thin and lossy metal segments (e.g., Ni, Rh, and Ru) synthesized via templated synthesis were found to effectively bridge the plasmonic oscillations of Au NR dimers [44,100]. Such bimetallic nanorods have tunable optical properties that are relevant for photocatalysis based on Rh or Ru photocatalysts [65,101]. The surface E-Field enhancement at the Ru or Rh segment can be especially enhanced when it is located at the end of the Au NR, while light absorption is the highest when the segment is in the middle of the Au NR. Such light absorption and surface E-field enhancement can be controlled independently from each other by controlling the location of the lossy metal segment within the bimetallic plasmonic NR (Figure 10i) [44]. In both cases, the LSPR wavelength and the wavelength at which the highest enhancement in the surface E-field and light absorption occurs can be tuned by varying the total length of the bimetallic NR [44].

Electrodeposition within porous alumina membranes can be routinely used to produce centimeter-sized metal NR arrays, which are well-suited for photoelectrochemical and photovoltaic experiments. Freestanding Au NR arrays capped with a thin (i.e., 20 nm) TiO_2_ layer and decorated with Pt NPs were one of the first successful plasmonic photocathodes used for water splitting [102]. The photocurrent was measured followed the extinction spectra of the Au NRs (Figure 10f), indicating that the hot charge carriers responsible for the hydrogen evolution reaction are generated through to the plasmon decay of the Au NRs and are injected through the Au–TiO_2_ Schottky barrier into the Pt catalyst and the electrolyte [102]. When an additional Co-based oxygen evolution catalyst was electrodeposited on the lower parts of the Au NR array, the device acted as an autonomous solar water splitter (Figure 10j). The H_2_ production of this device was significantly higher when irradiated with wavelengths > 520 nm compared to wavelengths between 310 nm and 520 nm, which showed that the highly energetic electrons responsible for the hydrogen evolution reaction are mostly generated due to the plasmon decay in the Au and not in the semiconductor [102].

Similarly, a free-standing Au NR was used as a photovoltaic cell. The NRs were conformally coated with TiO_2_ to separate them from the Ti contact on top of the cell. By measuring the photocurrent of the cell, it was shown that the photon-to-electron conversion efficiency has its maximum at the LSPR wavelength of the Au NRs, demonstrating electron–hole pair photogeneration within the Au NRs, followed by hot electron transfer through the Au/TiO_2_ Schottky barrier and charge extraction at the Ti contact [103].

#### 4.3.2. On-Wire Lithography

**Figure 11:**
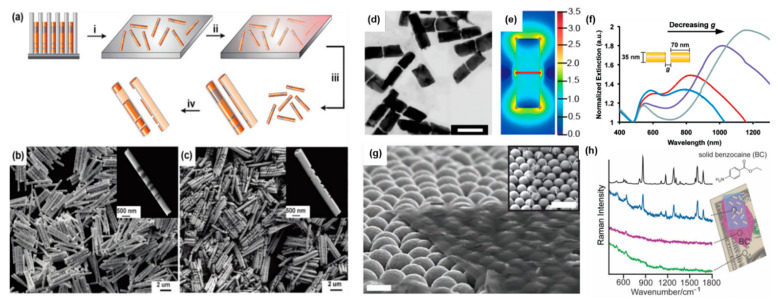
On-wire lithography. (**a**) OWL synthetic scheme describing (i) dispersal of multisegmented nanowires onto a substrate, (ii) deposition of a thin backing layer (i.e., SiO_2_) on the nanowires, (iii) sonication to disperse the wires back into solution, (iv) selective etching of sacrificial segments. (**b**,**c**) SEM images of Au–Ag nanowires before (**a**) and after (**b**) selective etching of sacrificial Ag segments. (**d**) TEM image of Au NR dimers. Scale bars are 100 nm. (**e**) Simulated E-field enhancement around a Au NR dimer with dimensions such as the one shown in (**d**) with a 785 nm excitation wavelength and polarization along NR length axis. Log scale for clarity. (**f**) Extinction spectra of Au NR dimers with different gap sizes. (**g**) SiO_2_ nanosheets with embedded Au NR dimers on SiO_2_ microspheres. Scale bars are 400 nm and 1 µm for (**g**) and the inset, respectively. (**h**) Detection of benzocaine on the surface of a dollar bill. The black spectrum is of crystalline benzocaine and the green spectrum is of the dollar bill without benzocaine. Spectrum with the nanosheets (blue) clearly shows Raman peaks arising from benzocaine; without the nanosheets no appreciable signal correlating to benzocaine can be seen (purple). (**a**) Reprinted with permission from ref. [24]. Copyright 2011 American Chemical Society. (**b**,**c**) From ref. [29]. Reprinted with permission from AAAS. Copyright 2005. (**d**,**e**) Reprinted with permission from ref. [107]. Copyright 2012 American Chemical Society. (**f**) Reprinted with permission from ref. [106]. Copyright 2011 American Chemical Society. (**g**,**h**) Reprinted from ref. [48]. Copyright 2012 Wiley-VCH.

Electrochemical templated synthesis provides the opportunity to control the metal segment lengths electrochemically down to the sub-2 nm regime. This was demonstrated by on-wire lithography (OWL), which can be used to synthesize one-dimensional arrays of metal NRs with well-defined nano- and micrometer scale gaps (Figure 11) [29]. Rather than preparing multisegmented nanowires with functional segments, OWL is based on the use of thin sacrificial segments that are used as placeholders to isolate the target material segments. During OWL [105], multisegmented nanowires are released into solution and dispersed on a substrate. For rather thin nanowires (e.g., with diameters < 100 nm), it is necessary to disperse the nanowires via vacuum filtration through a nanoporous membrane to avoid agglomeration. The dispersed multisegmented nanowires are then coated with a conformal inorganic backing layer (silicon oxide or silicon nitride). Subsequently, the coated nanowires are redispersed into solution and selectively etched, which yields well-defined one-dimensional nanorod arrays composed of the target material, which are held together by the backing layer (Figure 11a–c). The method can be used for making structures with diameters as small as 35 nm [106]. Using appropriate electrodeposition parameters, the gap length between the metal segments can be controlled down to the sub-2 nm regime [48]. OWL offers the possibility to fabricate Au NR dimers with reproducible and tailorable sub-2 nm gap sizes with a high yield. Even for gap sizes as small as 2 nm, 96% of the synthesized structures were gapped Au NR dimers with a typical standard deviation in segment and gap lengths of 10–15% [107]. Alternative approaches to synthesize gapped NP dimers either rely on complex synthetic steps or expensive techniques or result in less well-defined structures due to the rather uncontrolled aggregation of NPs. Thus, the spatial resolution and material compatibility afforded by OWL is beyond the current capabilities of state-of-the art lithography techniques, such as electron beam lithography. Remarkably, OWL can be routinely achieved using bench-top electrochemical methods. One of the advantages of OWL is its ability to generate solution dispersible arrays that are also stable in the solid state. The versatility of OWL has allowed for the fabrication of a wide variety of structures that have led to advances in organic electronics [108], SERS [48,107], plasmonics [43,99], and biosensing [109]. OWL can be used with a variety of metals as well as with some conjugated polymers, such as polythiophene [43].

Plasmonic applications:

The longitudinal LSPR of gapped metal NRs can be controlled by changing the total length of the NRs and the gap size [48,107]. At a constant metal segment length, increasing the gap size leads to a blue shift of the LSPR frequency due to a weaker coupling of the individual NR segments (Figure 11f) [106]. As such, OWL has allowed some complex mechanistic studies on the optical coupling of one-dimensional NR arrays [106]. The effect of distance on the plasmonic modulation of the emission band of a photoluminescent polymer (polythiophene) was studied and demonstrated as the proof-of-concept of a plasmophore ruler [43]. Additionally, the ability to generate plasmonic hot spots at desired wavelengths makes OWL a well-suited technique for fundamental research in SERS.

Indeed, the remarkable tailorability of OWL allows achieving large SERS enhancements at distinct excitation wavelengths. For Au NR dimers separated by 2 nm gaps with a longitudinal LSPR wavelength tuned for a Raman excitation source of 785 nm, single particle SERS enhancement factors around 6.8 × 10^8^ were measured, which arose from the large E-field enhancement in the gapped structure (Figure 11d,e) [107]. This is impressive and well-suited for single-particle SERS. However, to use of these gapped structures for more conventional SERS experiments, a spatially uniform E-field enhancement is required. Because the individual gapped NRs can agglomerate in solution during drying or migrate into geometrical cavities of the Raman substrate, an alternative approach to synthesize nanosheets with embedded NRs was developed (Figure 11g) [48]. Remarkably, the embedded NRs keep their position within the nanosheets, even when they are dried on a substrate, thus providing a homogenous SERS enhancement over the sheets. Thanks to their flexibility, the sheets can conform to complex topographies, yielding a uniform enhancement in the Raman signal over large areas on arbitrary textured surfaces (Figure 11g,h).

#### 4.3.3. Coaxial Lithography

**Figure 12:**
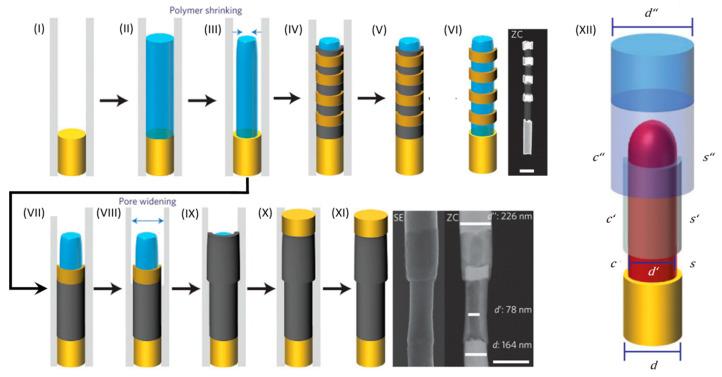
Coaxial lithography. (**I**–**V**) Scheme illustrating electrochemical synthesis of core–shell nanowires based on the controlled shrinking of the polymer core segment (shown in blue). (**VI**) Subsequent selective etching of sacrificial metal rings around the polymer core. ADF-STEM image of Au nanorings on a polypyrrole core. Scale bar is 500 nm. (**VII**–**XI**) Synthesis of core/shell/shell nanowires. After depositing shells around the polymer core, the pores of the tubular template are widened (**VIII**), and subsequent electrodeposition leads to the formation of core/shell/shell nanowires (**IX**). (**XI**) SE- and ADF-STEM image of a PANI core/Au ring/Ni shell nanowire. Scale bar is 250 nm. (**XII**) Scheme illustrating the geometrical and compositional parameters that can be controlled using COAL: diameters (d, d’, and d’’: from 20 nm to 400 nm), segment lengths (s, s’, and s’’: from 8 nm to a few micrometres) and compositions (c, c’, c’’, and polymers: PANI, PPy, PTh, and P3HT; metals: Au, Ag, Ni, Pt, and Pd; inorganic semiconductors: MnO2, CdSe, and CdS). Reprinted by permission ref. [30], Copyright 2015 Springer Nature.

OWL is exceptionally good at synthesizing one-dimensional arrays of metal nanostructures with well-defined dimensions. However, it cannot be used to control nanowire dimensions over the radial direction, which could help integrating plasmonic materials within functional semiconductor nanowires. Based on the concepts of OWL and the progresses made in the templated synthesis of core–shell nanowires, Mirkin et al. developed a method for synthesizing coaxial nanowires with sub-10 nm resolution in both the axial and radial dimensions, termed coaxial lithography (COAL) [30,110]. COAL allows for the synthesis of multi-compositional coaxial core/shell, core/multi-shell, and asymmetric nanowires via templated electrochemical deposition and selective wet-chemical etching processes (Figure 12).

After electrochemically depositing polymers within AAO membranes, it is possible to induce the contraction of the polymer via the evaporation of the solvent by exposing the membrane to vacuum conditions. Subsequent electrochemical deposition, as originally found by Whitesides and coworkers, leads to the synthesis of a conformal shell around the polymer core [111,112]. Similar to the synthesis of heterometallic segmented NRs, conformal multisegmented shells with well-defined lengths can be electrochemically grown around the polymeric core. After the dissolution of the membrane, the sacrificial shells can be selectively etched, yielding metal nanorings at well-defined positions around the polymeric core (Figure 12I–VI). This control in axial dimension is purely electrochemical and, thus, allows sub-10 nm resolution with relative size deviations in the 10% range.

Additionally, COAL can be used to control the radial dimensions of the resulting nanowires by widening the membrane pores using an aqueous NaOH solution. By adjusting the etchant concentration and etching time, the pore diameter and, thus, the radial dimension of the deposited shell can be controlled with a sub-5 nm resolution. Furthermore, control over the inner diameter of the shells and rings can be achieved by the controlled dissolution of the polymeric core. Thus, the inner and outer diameters of the shells can be adjusted by dissolving the polymeric core and the membrane pores, respectively. COAL has been successfully used to synthesize metal nanorings with a variety of different compositions, such as Au, Ag, Ni, Pt, and Pd, and well-defined geometric parameters. These nanorings can be located at well-defined positions around nanowires (Figure 12VI). In addition, it is possible to dissolve the polymer, while the nanorings are located at a fixed position within the inorganic membrane [30]. Alternatively, the AAO membrane and the polymeric core can be dissolved, yielding solution-phase nanoring dispersions [110].

**Figure 13:**
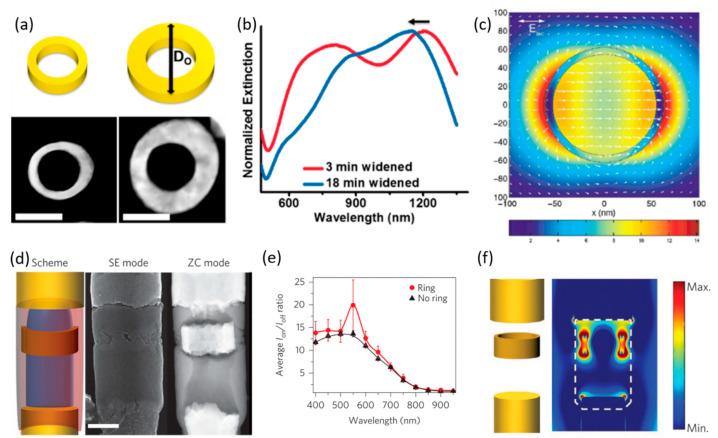
Tunable optical properties of Au nanorings. (**a**) Au nanorings with different outer diameters synthesized via COAL. Scale bar is 100 nm. (**b**) Normalized extinction spectra of the nanorings shown in (**a**). Large outer diameter in blue and small outer diameter in red. (**c**) Horizontal cross-section of the simulated E-field enhancement of a Au nanoring. E-field polarization along the nanoring diameter. (**d**) Scheme, SE, and Z-contrast STEM images of the P3HT core/CdSe shell nanowires with a Au ring. Scale bar is 100 nm. (**e**) I_on_/I_off_ ratios as a function of wavelength of nanowires with (red circles) and without (black triangles) a ring. (**f**) Simulated E-field intensity maps of the metal segments (nanowire shown in **d**) recorded at 532 nm (logarithmic scale). (**a**,**b**) Adapted from ref. [110]. Copyright 2015 American Chemical Society. (**c**) Reprinted with permission from ref. [46]. Copyright (2003) by the American Physical Society. (**d**–**f**) Adapted from ref. [30]. Copyright 2015 Springer Nature.

Plasmonic applications:

The optical properties of these nanorings are tunable by adjusting the geometric parameters (Figure 13a,b). Similar to NRs, increasing the nanoring length leads to a red shift of the most intense LSPR wavelength. Increasing the outer diameter and decreasing the inner diameter leads to a blue shift of the LSPR wavelength due to a more disk-like extinction behavior [110]. The large volumes with a homogenous E-field enhancement (Figure 13c) provided by these hollow nanostructures makes them particularly interesting for photocatalysis and sensing applications. Under the right conditions, subsequent pore widening can be used to grow a second shell around a previously prepared core/shell nanowire (Figure 12VII–XI). In addition to the remarkable geometric control of this technique, COAL is compatible with most materials that can be electrodeposited. Indeed, the polymeric core can be dissolved and replaced after the growth of the desired shells with a different core material, such as CdS, CdSe, MnO_2,_ or a metal. Thus, it is possible to synthesize quite a variety of core–shell nanowires via COAL. Figure 12XII schematically shows the geometrical parameters that can be tuned via COAL and presents the different materials that can be used. Such core–shell geometry can enhance charge separation, which can benefit organic semiconductors that suffer from short carrier diffusion lengths, such as, for example, poly(3-hexylthiophene) [112].

The high tailorability offered by COAL was demonstrated by the synthesis of a highly complex heterostructure composed of a p-type semiconductor core (P3HT) with a Au nanoring at a deliberate axial position and an n-type semiconductor (CdSe) shell that surrounds both the nanoring and the core. Both ends of this core–shell nanostructure are connected to a Au NR segment (Figure 13d). This structure is not only rather complex to fabricate in such a high yield but also enhances the photocurrent of the radial pn-junction due to the presence of the plasmonic Au nanoring (Figure 13e). FDTD simulations indicate that the E-field around the Au nanorings is largely enhanced at wavelengths around 530 nm. This E-field enhancement leads to an increased electron-hole generation rate, and, since the nanoring is only blocking a small area of the axial pn-junction, the overall photocurrent significantly increases compared to a radial pn-junction without the metal nanoring.

### 4.4. Silicon Nanowire Arrays (3DEAL)

While COAL offers many degrees of freedom to engineer core–shell nanowire architecture, it is limited to semiconductor materials that need to be grown from solution and is, thus, not compatible with more technologically relevant material systems, such as single-crystalline Si nanowires.

By combining the outstanding optoelectronic properties of Si nanowire arrays [113,114,115] with metal nanostructures, it is possible to access a range of attractive architecture with a wide variety of applications. The metal nanostructures can act either as catalysts [116,117,118,119], electrical contacts [120,121,122], or plasmonic materials [123,124,125,126]. Because of the high surface area of Si wire arrays, they can afford a higher loading of catalysts while limiting optical losses due to parasitic light absorption within the catalyst. Huskens et al. reported the functionalization of Si microwire array photocathodes with NiMo catalysts, which, under the proper loading conditions could lead to an unprecedented ideal regenerative cell efficiency > 10% [116]. Additionally, because metallic nanostructures can greatly enhance the incident electric field (E-field) under plasmonic excitation [2,4], their integration within Si nanowires can be used to control light absorption and emission processes in the nanowire. For example such hybrid structures have been used for SERS [127], ultra-thin photovoltaic cells [126], and efficient hybrid systems for water splitting [124,125]. By confining the strong plasmonic fields around Si nanowires, it is also possible to enable visible light photoluminescence and electroluminescence from Si [123,128], which is quite exceptional considering the fact that silicon is a poor light emitter due to its indirect band gap.

**Figure 14:**
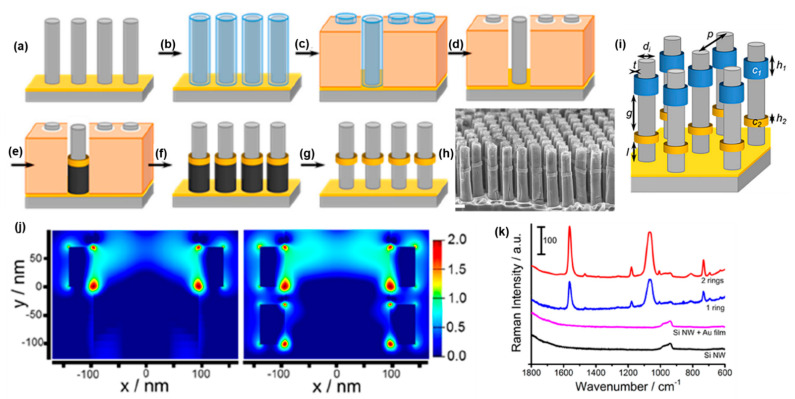
Three-dimensional electrochemical axial lithography (3DEAL). (**a**–**g**) Scheme showing the synthesis steps. The Si nanowires (in gray) are conformally coated with a SiO_2_ shell (in blue) and embedded within a polymer (orange). The dissolution of the SiO_2_ shell generates annular pores around the Si nanowires. The pores guide the electrodeposition of multisegmented shells around the Si nanowires, starting from the Au film at the bottom (here: nickel shown in black and Au in yellow). Dissolution of the polymeric membrane and selective etching of the sacrificial shell (here: nickel) leads to a well-defined metal shell (here: a Au ring). (**h**) Secondary electron SEM image of a Si microwire array with a Au ring. Scale bar is 1 µm. (**i**) Scheme showing the geometric parameters that can be controlled using 3DEAL, including the Si nanowire diameter and pitch, the outer diameter, height, and axial position of the nanorings. (**j**) E-field enhancement maps of regions of Si nanowires with one Au ring (left) and two Au rings (right). The incident light propagates along the y axis, and the electric field is polarized along the x axis. Excitation wavelength is 785 nm. The scale for the E-field enhancement is logarithmic. (**k**) Raman spectra of 1,4−benzenedithiol−functionalized Si nanowires (black curve), Si nanowires with a Au film at the bottom (magenta curve), a Si nanowire array with a single Au ring (blue curve), and a Si nanowire array with two Au rings spaced by a 30 nm gap (red curve). Reprinted with permission from ref. [31]. Copyright 2018 American Chemical Society. Further permission related to the material excerpted should be directed to the ACS.

However, creating hybrid architecture interfacing single-crystalline Si (c-Si) wire arrays with metal nanostructures is challenging. The most promising approaches to pattern specific areas of Si wire arrays to date have been electrochemical. For example, Huskens et al. reported the preferential (photo)electroreduction of metal ions either on the p-side or the n-side of axial n^+^/p microwires [129]. A different approach was developed by Lewis et al. to selectively deposit metal NPs either at the top of Si microwires or homogeneously distributed through the entire wire height by spatially controlling the photogeneration of charge carriers based on the irradiation wavelength during the photodeposition: blue-UV being mostly absorbed at the top of the wire due to the large absorption of Si in this range, while red photons are absorbed homogeneously through the wires due to the lower absorption in the red of Si [130]. However, the spatial resolution provided by these techniques is poor. Figure 14 presents the implementation of COAL concepts to perform three-dimensional lithography within vertically oriented single-crystalline Si nanowire arrays with a sub-10 nm spatial resolution in both the radial and axial directions, termed three-dimensional electrochemical axial lithography (3DEAL) [31].

Arrays of single-crystalline Si nanowires can be obtained using lithographic techniques and metal-assisted chemical etching (MACE) [131]. A patterned noble metal hole film is thereby coated on top of a Si wafer, e.g., with colloidal lithography [132]. During MACE, the reduction of H_2_O_2_ is catalyzed by the metal film, which injects holes into the silicon that dissolves in the presence of HF. Since this reaction is much slower on the bare Si, the metal films etch through the silicon wafer, producing highly ordered vertically aligned Si nanowire arrays (Figure 14a). Then, 3DEAL starts with the selective coating of the Si nanowires with a thin layer of SiO_2_, followed by the Si nanowire embedding within a non-conductive polymer film. The subsequent dissolution of the SiO_2_ creates annular pores around the nanowires. To ensure that the following electrodeposition starts from the bottom of the pores at the Au film, the Si nanowires are coated with an additional thin isolating SiO_2_ layer. Subsequently, the desired multisegmented shell structure can be electrodeposited within the annular pores. After dissolving the polymer, sacrificial materials are selectively etched, and the final structure is obtained. Figure 14h shows the geometric parameters that can be controlled via 3DEAL. The diameter and pitch of the nano- or microwires can be adjusted with the lithographic technique used to prepare the catalytic metal film. The SiO_2_ layer (Figure 14b) determines the outer diameter and, thus, the thickness of the conformal rings. The length, axial position, and composition of the nanorings is controlled via electrochemical deposition. As such, 3DEAL allows the precise decoration of Si nanowire arrays with metal nanorings of different compositions at specific axial locations with negative feature sizes, i.e., gaps down to 5 nm. By modifying 3DEAL, it is possible to passivate specific regions of the Si nanowire with an insulating layer of SiO_2_ and locate well-defined patches where the silicon nanowire is bare. These bare Si segments can then be used for the spatioselective electrochemical deposition of metal catalysts, such as Pt and NiMo, at specific locations along the nanowires. Thus, using this modified version of 3DEAL, it is possible to locate the metal catalysts at well-defined axial positions along Si nanowire arrays while ensuring a good electrical contact with the silicon [133]. These functionalized and passivating nanowire arrays were found to be photoelectrochemically active for the hydrogen evolution reaction [133].

Plasmonic application:

As can be seen in Figure 14j, the decoration of a Si nanowire with a Au ring leads to a large enhancement of the E-field in close vicinity of the Au ring, which is especially large at the interface between Si and Au. By locating a second plasmonic ring around the Si nanowires, separated by a 30 nm gap, the E-field enhancement is even more pronounced. Due to these large E-field enhancements at each Si nanowire–Au ring interface over the macroscopic areas (e.g., >1 cm^2^), the Si nanowire arrays are well-suited for SERS (Figure 14k). Because the Au rings also significantly enhance the E-field in the Si (Figure 14j) and, thus, light absorption, 3DEAL can be used to adjust light absorption within Si nanowire arrays. Overall, 3DEAL is a powerful technique to perform mechanistic studies within three-dimensional arrays of metal rings within silicon nanowire arrays, where the in-plane pitch (i.e., 2D) is fixed by the nanowire array periodicity and the axial pitch (i.e., 1D) is fixed by 3DEAL.

## 5. Conclusions

Electrochemical syntheses have the advantage of being cost-effective and easy to implement in the lab. Table 1 summarizes the strengths and limitations of the electrochemical techniques reviewed here. Template-free ECD is particularly well-suited to deposit metal NPs with well-defined surface densities, sizes, and shapes on conducting surfaces, while recent reports have demonstrated its ability to synthesize particularly complex metal nanostructures. This is highly relevant for both fundamental and applied research in plasmonics. Thanks to its simplicity, electrochemical Ostwald ripening has appeared as a remarkably powerful way to mass-produce commercial SERS substrates [70] and, as such, has a bright future for nanostructuring metals at a low cost. Similarly, electrochemical dealloying is amenable to the preparation of monolithic and large substrates that can be used for SERS. The use of ECD on patterned planar electrodes via LPNE and RIPPLE also has some potential for preparing 2D plasmonic arrays over large areas, which could have interesting new optical properties. Finally, templated approaches are particularly appealing: Indeed, COAL and 3DEAL allow the fabrication of complex 1-, 2-, and 3-dimensional nanostructures that cannot be obtained with any other technique, while the spatial resolution and yield provided by OWL is still unprecedented. Thus, the geometric control afforded by these electrochemical lithographic techniques is, to-date, unmatched. Overall, these electrochemical techniques only require standard bench-top lab equipment (e.g., a standard electrochemical potentiostat and a simple electrochemical cell) and are not time-consuming (the synthesis of the most complex structures can be achieved within less than 24 h). More conventional techniques, which offer a similar spatial resolution (e.g., e-beam lithography), are expensive, slow, cannot be used to pattern large surfaces, and are not able to pattern three-dimensional structures.

Because of the impressive synthetic control they provide, electrochemical lithographies have the potential to address some key questions in plasmonics. In that regard, OWL has been particularly successful at providing breakthroughs in both fundamental and applied research that involve plasmonic metal nanogaps. In addition to the already impressive work that has been conducted using OWL, COAL, and 3DEAL, we believe that there is lots of room for performing exciting fundamental studies that cannot be answered using conventional approaches in the area of plasmonic photocatalysis. The ability to couple different metals at the nanoscale in 1D, 2D, and 3D could be used to synthesize complex heterostructured nanoscale photocatalysts with well-defined and tunable optical and catalytic properties. The possibility to engineer plasmonic and photonic coupling within 3D plasmonic arrays also offers a range of opportunities for performing SERS studies in 3D. As such, templated ECD techniques are very well-suited for complex mechanistic studies in SERS, plasmonics, and photocatalysis.

## Figures and Tables

**Table 1:** Advantages and limitations of the electrochemical syntheses discussed in this Review.

Technique	Advantages	Limitations	Spatial Resolution	Size Dispersity	References
ECD	Cost-effective and large scale Synthesis of NPs with complex morphologies Can improve our understanding of colloidal syntheses	Low yields compared to conventional wet-chemical colloidal syntheses	N.A.	<10–15%	[54,61,62,63,64]
Ostwald electrochemical ripening	Cost-effective Simple and large-scale Synthesis of a high density of nanoscale gaps/plasmonic hot spots	No precise control over interparticle distances and NP sizes	Nanometer-sized gaps	Rather large	[67,70,72]
Electrochemical dealloying	Cost-effective Simple and large-scale Can form porous metal thin films.	No precise control over distances between the nanoscale building blocks	Sub-50 nm features	Rather large	[74,76,78]
Electrochemical reduction of NZVN	Cost-effective Large-scale synthesis of Au, Ag, and Cu nanostructures with various geometries	Not suited to synthesize well-defined monodisperse nanostructures	N.A.	Rather large	[79,80,81,82,83]
ECD on patterned planar electrodes	Synthesis of metal/semiconductor nanowires with mm-scale lengths Synthesis of 2D nanoring arrays	Limited in throughput and accessibility by the patterning step or the surface topography of the surface used for ECD	Height: ca. 5 nm Width: ca. 40 nm	<10–15%	[54,87,88,89,90]
ECD within porous membranes	Large-scale Synthesis of multisegmented nanowires with controllable segment lengths. Can be used to prepare vertically oriented nanowire arrays Well-suited for plasmonics (pore diameters < 100 nm)	Requires a porous membrane The radial dimension is fixed by the membrane pore size	Sub-5 nm in axial dimension	<10–15%	[44,91,92,93,94,95,96,97,98,99,100,101,102,103,104]
OWL	Large-scale Precise synthesis of nm-sized gaps Synthesis of 1D gapped nanowire arrays	Requires a porous membrane The radial dimension is fixed by the membrane pore size	Sub-5 nm in axial dimension	<10–15%	[29,43,48,105,106,107,108,109]
COAL	Synthesis of metal nanorings with tunable dimensions Synthesis of core/multishell nanowires Synthesis of asymmetric nanowires	Requires a porous membrane Limited to materials that can be grown from solution (i.e., usually multicrystalline low-quality semiconductors)	Sub-10 nm in axial and radial dimensions	<10–15%	[30,110,111,112]
3DEAL	Large-scale Compatible with single-crystal Si Can be used to pattern pre-synthesized Si microwire and nanowire arrays with well-defined metal nanorings and NPs at specific axial locations	To date, it requires a conducting metal film at the bottom of the wire array, which can be provided via metal-assisted chemical etching	Sub-10 nm in axial and radial dimensions	<10–15%	[31,113,114,131,132,133]
